# Perturbing the Hypothalamic–Pituitary–Adrenal Axis: A Mathematical Model for Interpreting PTSD Assessment Tests

**DOI:** 10.1162/CPSY_a_00013

**Published:** 2018-02-01

**Authors:** Lae Un Kim, Maria R. D’Orsogna, Tom Chou

**Affiliations:** 1 Department of Biomathematics, University of California, Los Angeles, USA; 2 Department of Mathematics, California State University, Northridge, USA

**Keywords:** HPA axis, PTSD, cortisol, DEX suppression test, ACTH challenge test

## Abstract

We use a dynamical systems model of the hypothalamic–pituitary–adrenal (HPA) axis to understand the mechanisms underlying clinical protocols used to probe patient stress response. Specifically, we address dexamethasone (DEX) and ACTH challenge tests, which probe pituitary and adrenal gland responses, respectively. We show that some previously observed features and experimental responses can arise from a bistable mathematical model containing two steady-states, rather than relying on specific and permanent parameter changes due to physiological disruption. Moreover, we show that the timing of a perturbation relative to the intrinsic oscillation of the HPA axis can affect challenge test responses. Conventional mechanistic hypotheses supported and refuted by the challenge tests are reexamined by varying parameters in our mathematical model associated with these hypotheses. We show that (a) adrenal hyposensitivity *can* give rise to the responses seen in ACTH challenge tests and (b) enhanced cortisol-mediated suppression of the pituitary in subjects with PTSD is not necessary to explain the responses observed in DEX stress tests. We propose a new two-stage DEX/external stressor protocol to more clearly distinguish between the conventional hypothesis of enhanced suppression of the pituitary and bistable dynamics hypothesized in our model.

The hypothalamic–pituitary–adrenal (HPA) axis is a neuroendocrine system that regulates the secretion of cortisol by the adrenal cortex in response to stressors. To understand how the HPA axis functions, especially in the context of posttraumatic stress disorder (PTSD), a number of challenge tests have been developed. These tests typically involve measuring changes in key endogenous hormone levels after the administration of their synthetic analogues in both PTSD and normal subjects. In this article, we reinterpret the outcomes and dynamics of some of the challenge tests through mechanistic models of HPA axis dynamics.

Cortisol (a type of glucocorticoid steroid hormone) is a “stress hormone” that regulates or supports a variety of important cardiovascular, metabolic, immunologic, and homeostatic functions (Watson, Brüne, & Bradley, 2016). As is typical for hormones, maintaining cortisol concentrations within appropriate ranges during both stress response and in the basal state is essential for normal physiological function. A stress response is typically initiated when neurons in the paraventricular nucleus (PVN) of the hypothalamus receive increased synaptic inputs from various regions of the brain, each containing information about certain types of stressors. These synaptic inputs induce the PVN neurons to release corticotropin-releasing peptide hormone (CRH) into the portal blood vessel connecting the hypothalamus to the anterior pituitary. Released CRH travels to the anterior pituitary and activates the secretion of adrenocorticotropin hormone (ACTH). ACTH travels via the bloodstream to the adrenal cortex, located above the kidneys, where it stimulates cortisol secretion. Finally, cortisol travels back to both the pituitary and the hypothalamus to suppress their activities, completing the negative feedback loop and returning cortisol to a basal level. Both ACTH and cortisol exhibit ultradian (hourly) and circadian (daily) oscillations. The basic interactions regulating the HPA axis dynamics are summarized in Figure 1. If the HPA axis is dysregulated, once stimulated, cortisol may fail to return to basal levels, disrupting other functions and causing comorbidities. For example, excessive cortisol is associated with major depressive disorder (MDD; Gold & Chrousos, 2002), while low cortisol is generally reported among PTSD patients. Clini cal observations confirm lower than normal cortisol levels in the urine of PTSD patients col lected over a 24-hour period (Mason, Giller, Kosten, Ostroff, & Podd, 1986; Yehuda, Kahana, Binder-Brynes, Southwick, et al., 1995; Yehuda et al., 1990) and in blood samples drawn at 15-min intervals over 24 hours (Bremner, Vermetten, & Kelley, 2007). In one measurement, cortisol levels collected in urine of PTSD subjects were 40.9 ± 12.3 *μ*g/day and appreciably lower than the 62.8 ± 22.2 *μ*g/day collected in urine of normal subjects (Yehuda et al., 1990). Blood samples also showed that plasma cortisol levels were consistently lower among PTSD patients over the 24-hour period and significantly lower in the afternoon (Bremner et al., 2007).

**Figure 1.**
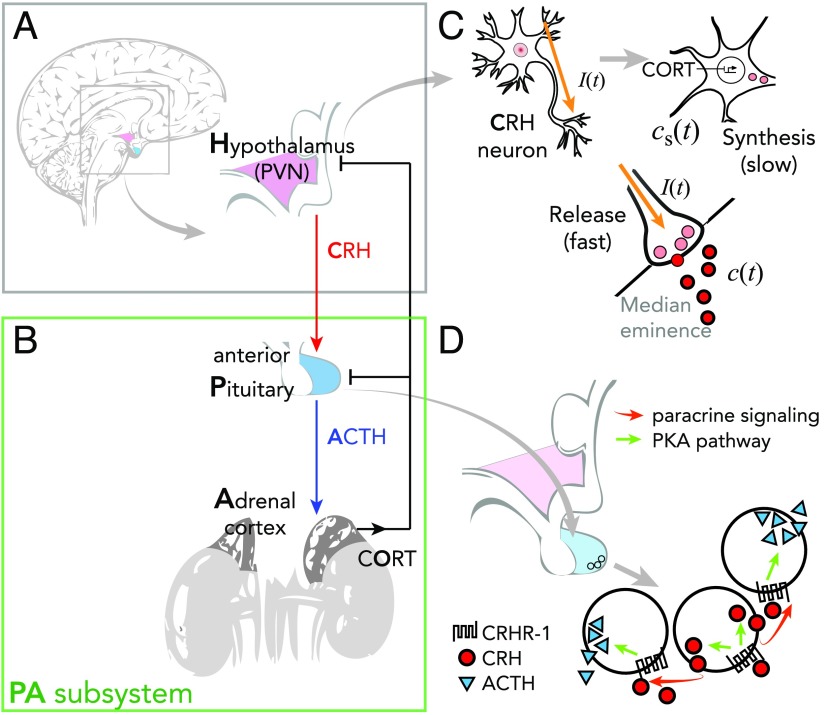
**Schematic of the HPA axis.** A) Stress is processed in the central nervous system and a signal is relayed to the PVN in the hypothalamus to activate CRH secretion into the hypophyseal portal system. B) CRH diffuses to the pituitary gland and activates ACTH secretion. ACTH travels down to the adrenal cortex to activate cortisol (CORT) release. Cortisol inhibits both CRH and ACTH secretion to downregulate its own production, forming a closed loop. C) Negative feedback of cortisol suppresses CRH synthesis in the PVN, ultimately reducing the amount of stored CRH and its subsequent release. External inputs, such as stressors and circadian inputs, directly affect the release rate of CRH at the axonal terminal. D) CRH released by the PVN stimulates the protein kinase A (PKA) pathway to activate release of CRH by the anterior pituitary, contributing to ACTH secretion in an auto/paracrine fashion.

Despite the general trend of lower cortisol under PTSD, a few contradicting reports may be found in the literature. Most of them are surveyed in a recent meta-analysis (Meewisse, Reitsma, Vries, Gersons, & Olff, 2007). For example, urine measurements in Rasmusson et al. (2001) showed a marginally significant increase of cortisol in premenopausal women with PTSD; the basal cortisol level in Yehuda, Golier, Yang, and Tischler (2004a) showed no difference between PTSD and control groups. The overall pooled estimate of the mean cortisol level from the meta-analysis (Meewisse et al., 2007) indicated that cortisol was lower within the PTSD group (standardized mean difference =−0.12 with *p* value 0.24) but concluded that the difference was not significant. On the other hand, the difference between PTSD and control subjects was shown to be significant in some subgroups, such as females and those exposed to certain types of trauma (Meewisse et al., 2007). Most importantly, the meta-analysis found that cortisol levels were significantly lower among PTSD patients when compared to subjects who had not been previously exposed to trauma (standardized mean difference =−0.35 with *p* value 0.007).

Another, more recent, meta-analysis (Morris, Hellman, Abelson, & Rao, 2016) studied the relationship between cortisol levels measured within 72 hours from trauma exposure and subsequent PTSD, symptoms. Overall, lower cortisol levels were shown to be associated with PTSD but not in a statistically significant way (overall correlation *r* = −0.07 with *p* value 0.449). However, as with the Meewisse meta-analysis (Meewisse et al., 2007), cortisol levels for subjects aged 30 years or older showed a significant negative correlation to PTSD (correlation *r* = −0.19 with *p* value 0.053). Lower cortisol levels may be associated with PTSD for other subgroups. One of the goals of our study is to propose a theory to explain how trauma exposure can lead to altered cortisol dynamics and understand why certain subgroups are likely to express lower posttraumatic cortisol levels.

Previous mathematical models (Andersen, Vinther, & Ottesen, 2013; S. Gupta, Aslakson, Gurbaxani, & Vernon, 2007) also attributed alterations in cortisol levels to bistability but did not consider ultradian oscillations known to arise in HPA dynamics. Alternatively, Gudmand- Hoeyer, Timmermann, and Ottesen (2014), Sriram, Rodriguez-Fernandez, and Doyle (2012), and Bangsgaard and Ottesen (2017) attributed altered levels in observed cortisol levels in PTSD and depression to changes in parameter values. In particular, Bangsgaard and Ottesen (2017) recently found that their mechanistic model yielded good fits to measured cortisol and ACTH levels of depressed and control subjects, both individually and as a group, and identified parameters relevant in diagnosing depression. Other models have focused on showing whether endogenous ultradian oscillations are allowed in a class of models of the HPA axis (Savić, Jelić, & Burić, 2006; Vinther, Andersen, & Ottesen, 2011) or have investigated the feedback mechanism between the HPA axis and the memory system (Savić, Knežević, & Opačić, 2000). Further discussion on the structure and features of previous mathematical models can be found in Kim, D’Orsogna, and Chou (2016) and Bertram (2015).

There is no consensus on which mathematical model is mechanistically the most accurate. Hosseinichimeh, Rahmandad, and Wittenborn (2015) selected five publications (Andersen et al., 2013; Conrad, Hubold, Fischer, & Peters, 2009; Jelić, Smiljana, Čupić, & Kolar-Anić, 2005; Sriram et al., 2012; Vinther et al., 2011) and compared model predictions with cortisol and ACTH levels obtained from 17 healthy individuals. Measured ACTH levels were substituted into the equation for cortisol for each of the models, and cortisol data were generated. Later, the role of cortisol and ACTH was reversed: Measured cortisol levels were used to numerically determine ACTH. Simulated data and actual measurements were then compared. According to Hosseinichimeh et al. (2015), none of the five models analyzed provided a good fit with data: The best fitting model, by Andersen et al. (2013), still yielded a difference of an order of magnitude in cortisol between simulations and data. Moreover, Vinther et al. (2011) and Andersen et al. (2013) proved that oscillations could not arise within a class of mathematical models, including their own, without the inclusion of additional mechanisms.

Attempts at better calibrating the model for a more accurate description of the circadian rhythm yielded some improvement, with the mean percentage error decreasing from 94% to 66%. It is important to note that when computing the goodness of fit on the data of 17 subjects, parameters were fixed at the original values provided by the authors. Individual differences and/or variations in environments were not taken into account. These attempts at matching models with actual data show how challenging the task actually is. Many variables must be taken into account, including personalized parameter sets, circadian and ultradian rhythms, external inputs, and other factors, some of which may still be unidentified.

The focus of our work is not to exactly match data to a model but rather to provide a mechanistic framework to better understand the interplay between various components of the HPA axis and its overall qualitative behaviors. Our goal is to gain insight into how variations in parameters may affect the HPA equilibrium for each individual and how transitions may be induced between multiple equilibria if they exist. Moreover, truly validating our model would require extensive data measured both before and after exposure to trauma. Such data do not exist to the best of our knowledge, and we hope that our work could motivate new, specific measurements that may yield a complete picture of the HPA axis and its dynamics.

Previously, we developed a dynamical model of the HPA axis to describe the interactions among the key hormones mentioned earlier and the glucocorticoid receptor (GR) that mediates feedback activity of cortisol (Kim et al., 2016). In the current work, we use and adapt this model to specifically consider perturbations representing externally or internally induced changes as well as currently employed challenge test protocols and methodologies. For appropriate sets of parameters, our model exhibits bistability with two attracting limit cycles over which cortisol and ACTH oscillate with an ultradian (hourly) rhythm. Of the two distinct oscillating states, the one with lower averaged cortisol level was characterized as a diseased state, and the one with higher cortisol level was associated with the normal state. Within our model, prolonged stress-induced secretion of CRH can trigger transitions between normal and diseased states, suggesting a possible mechanism leading to the emergence of a low cortisol state after a traumatic experience. Within our previous analysis, all parameters were fixed, except for the one representing the net synaptic input experienced by the PVN neurons. In reality, many of the parameters may vary due to aging (D. Gupta & Morley, 2014), life experience (Dinces, Romeo, McEwen, & Tang, 2014), gender differences (Seeman, Singer, Wilkinson, & McEwen, 2001; Uhart, Chong, Oswald, Lin, & Wand, 2006), the use of medication (Simunkova et al., 2008), and especially in response to injuries. For example, selective serotonin reuptake inhibitor (SSRI) treatment was shown to increase hippocampus volume (Thomaes et al., 2014). This may in turn modulate the tonic inhibition exerted by the hippocampus on the PVN and subsequently change the baseline synaptic input to CRH neurons in the PVN. On the other hand, traumatic brain injury (TBI) can produce diffuse axonal injuries (DAI; Sharp, Scott, & Leech, 2014) that can affect the synaptic strength or activation pattern of the hypothalamus (McCullers, Sullivan, Scheff, & Herman, 2002). Recent studies on the influence of maternal HPA function in neonatal rats have suggested that early life experience can have long-lasting impacts on responsivity of HPA axis activation (Dinces et al., 2014). Finally, the strength of the negative feedback of cortisol in the pituitary is hypothesized to be enhanced under PTSD (Yehuda, Halligan, Grossman, Golier, & Wong, 2002). These examples are only a few of many conditions that may be associated with anatomical/physiological changes that can be interpreted, and thus incorporated, as alterations in the parameters of our model (Kim et al., 2016).

For a more accurate description of the physiology in the current work, we include the self-upregulation property of CRH through the auto/paracrine function of pituitary cells. This modification of the original model does not change its overall qualitative features. A detailed comparison between the original model (Kim et al., 2016) and the current model is included in Kim et al. (2017, Appendix B). Here we briefly analyze the nullcline structure of the new model as a function of parameters and analyze the conditions under which normal–diseased bistability arises, whether or not stress-induced transitions are possible between the bistable states and whether or not the experimentally observed ultradian oscillations can be reproduced. We also systematically investigate how the dynamics depend on relevant parameter changes associated with, for instance, cerebral ischemia. Understanding how changes in parameters affect transient and long-term behavior of the HPA axis could guide therapeutic strategies.

We specifically include perturbations that represent existing pharmacological challenge tests used to assess HPA axis function. For example, dexamethasone (DEX) has been used as an exogenous steroid that suppresses ACTH release in the pituitary. DEX suppression tests have shown that the relative decrease in cortisol is greater in PTSD patients than in those in a control group. The current viewpoint is that the greater decrease in cortisol seen in PTSD subjects is due to an enhanced negative feedback of cortisol. Within a mathematical framework, this effect would typically be modeled by changing the physiological parameters describing the negative feedback. Our model allows for a novel mechanism: Cortisol suppression may be due to *transitions* between bistable steady states without necessarily invoking parameter changes. In this picture, disruptions to the HPA axis could be alleviated by externally controllable inputs that lead to transitions between the bistable states rather than by permanently altering specific physiological parameters.

Finally, we demonstrate how our mathematical analysis can help address previously unresolved observations of challenge experiments. ACTH stimulation tests performed to assess adrenal sensitivity in PTSD subjects showed slightly increased cortisol response to ACTH administration among PTSD subjects (Radant et al., 2009). This observation contradicts the authors’ speculation of decreased adrenal sensitivity in PTSD subjects. We show that this experimental result may be in fact *consistent* with the decreased adrenal sensitivity hypothesis and emphasize that the interplay between all components of the HPA axis must be taken into account to fully understand its overall dynamics.

## Model and Methods

Our nondimensionalized model of the HPA axis based on the interactions shown in Figure 1 consists of a system of delay differential equations, as follows:

$$\frac{\text{d}c\text{s}}{\text{d}t}=\frac{1}{t_{c}}(\underset{c_{\infty }(o)}{\underset{︸}{\left({\overset{-}{c}}_{\infty }+e^{-bo}\right)}}-c\text{s}),$$ (1)

$$\frac{\text{d}c}{\text{d}t}=q_{0}I(t)\underset{h(c\text{s})}{\underset{︸}{\left(1-e^{-kc\text{s}}\right)}}+\underset{g_{c}(c)}{\underset{︸}{g_{\text{c,max}}\frac{{\left(q_{1}c\right)}^{n}}{1+{\left(q_{1}c\right)}^{n}}}}-q_{2}c,$$ (2)

$$\frac{\text{d}a}{\text{d}t}=c\underset{f_{a}(or)}{\underset{︸}{\frac{1}{1+p_{2}(or)}}}-p_{3}a,$$ (3)

$$\frac{\text{d}r}{\text{d}t}=\underset{g_{r}(or)}{\underset{︸}{\frac{{\left(or\right)}^{2}}{p_{4}+{\left(or\right)}^{2}}+p_{5}}}-p_{6}r,$$ (4)

$$\frac{\text{d}o}{\text{d}t}=a(t-t\text{d})-o,$$ (5)

where *c*s represents the amount of *stored* CRH at the axonal terminal of CRH secreting neurons in the PVN, *c* is the level of circulating CRH, *a* defines the level of circulating ACTH, *r* describes the level of available glucocorticoid receptor in the anterior pituitary, and *o* is the level of circulating cortisol. In Equations 3 and 4, the cortisol–receptor complex is assumed to form and dissociate under fast dynamics, and this level will be approximated as that of steady state by the product *o* × *r*. All parameters are listed in Table 1.

**Table 1.** Parameters and their effects on nullcline structure

**Parameter**	**Description**	**Effects on nullclines (when increased)**
*q*_0_	maximum release rate of CRH in basal state	an upward shift of the upper branch and a leftward shift of both knees of the *c*-nullcline

*q*_1_	circulating CRH for half-maximum self-upregulation	an upward shift of the upper branch and a leftward shift of both knees of the *c*-nullcline

*q*_2_	ratio of CRH and cortisol decay rates	a downward shift of the upper branch and a rightward shift of both knees of the *c*-nullcline

*g_c,max_*	maximum auto/paracrine effect of CRH in the pituitary	a rightward shift of the lower and upper knees of the *c*-nullcline and an upward shift of the upper branch

*n*	Hill coefficient of *g_c_*(*c*) describing the self-upregulation of CRH	a leftward shift of the left knee and a rightward shift of the right knee of the *c*-nullcline

*k*	relates stored CRH to CRH release rate	a leftward shift of the middle branch of the *c*-nullcline and an upward shift of the lower branch of the *c*-nullcline

*b*	relates cortisol to stored CRH level	a leftward shift of the middle branch of the *c*s-nullcline

*p*_2_	(*or*)-complex level for half-maximum negative feedback	a rightward shift and elongation of the oscillatory regime of the *c*s-nullcline

*p*_3_	ratio of ACTH and cortisol decay rates	a rightward shift and elongation of the oscillatory regime of the *c*s-nullcline

*p*_4_	(*or*)-complex level for half-maximum positive feedback on *r* production	elongation of the oscillatory branch of the *c*s-nullcline

*p*_5_	basal GR production rate by pituitary	shortening of the oscillatory branch of the *c*s-nullcline

*p*_6_	ratio of GR and cortisol decay rates	a rightward shift and shortening of the oscillatory branch of the *c*s-nullcline

*t*_*d*_	delay in the adrenal cortex in response to ACTH	elongation of the oscillatory branch of the *c*s-nullcline (Walker, Terry, & Lightman, 2010).

The introduction of *c*s is the most distinctive feature of our model in comparison to others (Andersen et al., 2013; S. Gupta et al., 2007; Sriram et al., 2012; Walker et al., 2010) and allows us to more realistically model aspects of CRH dynamics that occur on different timescales. The two variables, *c*s and *c*, distinguish the two stages of the CRH secretion process: (a) the “slow” synthesis and packaging process of CRH peptides as regulated by cortisol and (b) the “fast” release process of CRH into the median eminence governed by synaptic activities and nongenomic effects of cortisol. The constant *t*_c_ reflects the slow timescale (minutes to hours) over which the amount of stored CRH, *c*s, relaxes toward a target value $c_{\infty }(o)$ relative to the timescale of CRH release (milliseconds). The target value $c_{\infty }(o)$ is set by circulating cortisol levels *o*(*t*) and embodies its negative feedback on CRH synthesis.

The two state variables, *c*s and *c*, are coupled by Equation 2 through a saturating and monotonically increasing function *h*(*c*s) = 1 − *e*^−*kc*s^ so that the average release rate of CRH increases with more stored CRH available. Self-upregulation of CRH release by the hypothalamic PVN neurons was included in our previous model (Kim et al., 2016) and was based on an experiment (Ono, Castro, & McCann, 1985) that showed that when CRH is injected into the third ventricle, PVN neurons increase their rate of CRH release into the median eminence. Thus this experiment indicates self-upregulation only if the CRH released in the median eminence directly and immediately increases the CRH level in the cerebrospinal fluid (CSF) filling the third ventricle. This is certainly a plausible mechanism. Yet, because a direct connection between the two pools of CRH is not yet well established, we improve our model by considering another relevant source of CRH activity.

Other measurements (Giraldi & Cavagnini, 1998) have demonstrated that CRH is also produced and secreted by the pituitary itself and that ACTH secretion in the anterior pituitary is upregulated in an auto/paracrine fashion by CRH secretion. We correspondingly revise the model presented in Kim et al. (2016) and in Equation 2 of the current work to include both CRH secretion due to the stimulation of the PVN neurons, as modulated by the synaptic input *I*(*t*) and the amount of stored CRH *c*_*s*_, and CRH secretion due to the auto/paracrine activity of the anterior pituitary, as described by the increasing Hill-type function *g*_*c*_(*c*). Its amplitude *g*_c,max_ can be estimated by isolating the self-upregulated CRH activity of the pituitary (Giraldi & Cavagnini, 1998). Together with the decaying term, our new Equation 2 describes the time rate of change of the total CRH concentration that can influence the anterior pituitary.

To include changes in synaptic input under stress, *I*(*t*) is modeled as a time-dependent parameter *I*(*t*) = *I*_0_ + *I*_ext_(*t*), where *I*_0_ is the minimum basal input level and *I*_ext_(*t*) is the increase in the synaptic input induced by external stress.

The concentrations of ACTH *a*, cortisol in circulation *o*, and the availability of glucocorticoid receptor GR in the pituitary *r* obey Equations 3, 4, and 5, respectively, and comprise the pituitary–adrenal (PA) subsystem, in which *c* can be viewed as a control parameter. The nonlinear multistate dynamics are defined by the decreasing and saturating regulation terms *f*_*a*_(*or*) and *g*_*r*_(*or*), respectively. The characteristic timescale constant *t*_*d*_ is normalized by the decay rate of cortisol and is proportional to the time required for cortisol synthesis and release by the adrenal cortex after stimulation by ACTH. More details of the model, its dimensional form, and the choice of the functional forms used in Equations 1–5 can be found in Appendix A of Kim et al. (2016). A comprehensive list of all parameters and their descriptions is included in Table 1.

Walker et al. (2010) showed that the PA subsystem (Equations 3–5) exhibits a limit cycle for a range of fixed time delay, *t*_d_. For concreteness, we consider a time delay of 15 min (in dimensionless units, *t*_d_ = 1.44) for the rest of this article. Moreover, the amplitude and the frequency of the limit cycle were shown to depend continuously on *c*, so that the limiting behavior of the PA subsystem could be unequivocally determined by the value of *c*. The separation of the two timescales—the faster timescale of the (*a*,*r*,*o*) limit cycle in the PA subsystem and the slower timescale governing the CRH synthesis process—allows us to study the dynamics of the entire system (Equations 3–5) by confining our analysis to the reduced system on the (*c*s,*c*)-phase plane. This means that the long-term behavior of the entire system can be characterized by the structures of the nullclines of *c*s and *c* projected onto the (*c*s,*c*)-plane. In particular, for certain sets of parameters, the nullcline structure exhibits bistable fixed points on the (*c*s,*c*)-plane that can be characterized as the diseased and normal modes of the PA subsystem, each marked by ultradian oscillations and distinct mean cortisol levels.

In the following sections, we outline the analysis of the present model described by Equations 1–5.

### Parameter Dependencies

In this section, we investigate how the model (Equations 1–5) behaves as their parameters are varied. This is important because some of the ones used in previous studies (Kim et al., 2016; Walker et al., 2010) were estimated from insufficient data or arbitrarily selected. We first examine the robustness of the long-term behavior of our model to changes in individual parameters. Because the long-term behavior of the model can be characterized by the nullcline structures of Equations 1–5 projected onto the (*c*s,*c*)-plane, in the following subsections, we study how parameter changes affect the *c*s- and *c*-nullclines. Descriptions of each parameter and their effects on the nullclines are listed in Table 1.

### Nullcline Analysis: *c*-Nullcline

The *c*-nullcline is defined as the set of (*c*s,*c*) that satisfies the following relation (obtained by setting Equation 2 equal to zero):

$$0=q_{0}Ih(c_{s})+g_{c}(c)-q_{2}c,$$ (6)

where

$$h(c\text{s})=1-e^{-kc\text{s}}\;\text{and}\;g_{c}(c)=\frac{g_{\text{c, max}}{\left(q_{1}c\right)}^{n}}{1+{\left(q_{1}c\right)}^{n}}.$$ (7)

**Figure 2.**
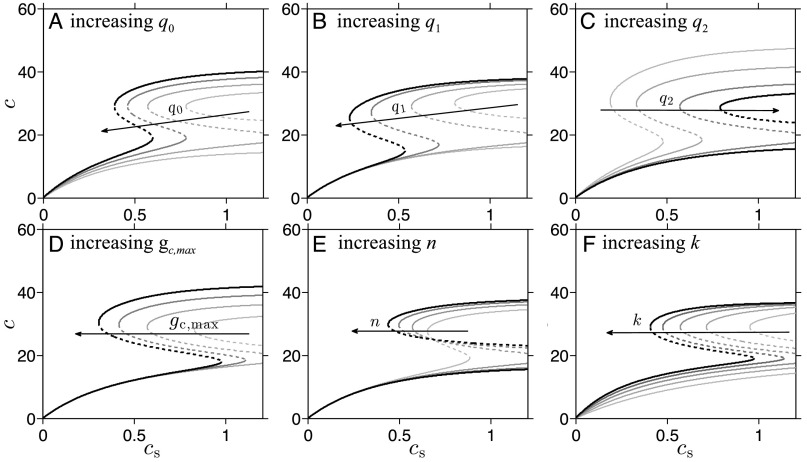
**Effects of changing parameters on the *c*-nullcline.** One of the nondimensionalized six parameters that affect the *c*-nullcline is varied over a range of values (from 80% to 120% of the reference values), and the corresponding *c*-nullclines are plotted. The dashed segment of the nullcline indicates the unstable steady states. Darker curves (in the direction of the arrows) are associated with greater values of the corresponding parameter. When not varied, parameters are set to the reference values *q*_0_ = 28.0(*I* = 1),*q*_1_ = 0.04,*q*_2_ = 1.8,*g*_c, max_ = 42,*n* = 6, and *k* = 2.83. A) The value of *q*_0_ is varied from 22.4 to 33.6. B) The value of *q*_1_ is varied from 0.032 to 0.48. C) The value of *q*_2_ is varied from 1.44 to 2.16. D) The value of *g*_c,max_ is varied from 33.6 to 50.4. E) The value of *n* is varied from 1 to 8. A saddle-node bifurcation occurs between *n* = 4 and *n* = 5. F) The value of *k* is varied from 2.26 to 3.40.

There are a total of seven parameters in the constraint defining the *c*-nullcline in Equation 6: *q*_0_,*q*_1_,*q*_2_,*I*,*g*_c,max_,*n*, and *k*. Our first approach is to study how time-dependent changes in the synaptic input *I* affect the dynamics of the system. Because *I* and *q*_0_ form a product, changes in *I* and *q*_0_ are equivalent; therefore we only consider changes *q*_0_,*q*_1_,*q*_2_,*μ*_*c*_,*n*, and *k*. In Figure 2, we vary one parameter at a time, while all others are kept fixed at nondimensional reference values *q*_0_ = 28.0,*q*_1_ = 0.04,*q*_2_ = 1.8,*I* = 1,*g*_c,max_ = 42,*n* = 5, and *k* = 2.83, as detailed in Kim et al. (2016) and in Appendix A of Kim et al. (2017).

Increasing *q*_0_ narrows and shifts the bistable region in the (*c*s,*c*)-plane toward lower values of *c*s, while extending the nullclines to larger values of *c* (Figure 2A). When *q*_1_ is increased, the bistable region also narrows and shifts toward lower *c*s values, but the range of the corresponding *c* values on the nullclines does not change significantly (Figure 2B). Increasing *q*_2_ (Figure 2C) appears to have the opposite effect: The bistable region is widened and the range of corresponding *c* values decreases. This behavior is expected, because it can be shown that the roots of Equation 6 depend on the ratio *q*_0_/*q*_2_. When *g*_c, max_ is increased, the upper branch of *c*-nullcline shifts toward higher values of *c*, and the bistable regime moves toward the lower *c*s values (Figure 2D). The Hill coefficient *n* of *g*_*c*_(*c*) (in Equation 2) also exhibits a saddle-node bifurcation at a critical point 4 < n^*^ < 5 (Figure 2E). Once bistability emerges, the upper and lower knees of the *c*-nullcline shift toward lower and higher values of *c*s as *n* increases. The bistable regime in *c*s is elongated as the two knees shift in the opposite direction from each other. Lastly, increasing *k* (Figure 2E) shifts the bistable region toward lower *c*s values without appreciably changing the values of *c* over which bistability exists.

By understanding how each parameter affects the *c*-nullcline, we can identify and predict which parameter changes disrupt HPA axis function. For example, we predict that increasing *k* will make the lower cortisol state less accessible because the range of *c*s covered by the lower branch of the *c*-nullcline narrows as *k* increases. Furthermore, we can use our results to interpret experimental reports of perturbations to the HPA axis. Any physiological disruption observed or associated with changes in long-term HPA function can be mapped to relevant parameter changes in our model.

### Nullcline Analysis: *c*s-Nullcline

The *c*s-nullcline is defined as the set of (*c*s,*c*) that satisfies the relation (obtained by setting Equation 1 equal to zero)

$$0=c_{\infty }(o)-c_{\text{s}}.$$ (8)

Equation 8 does not directly relate *c*sto *c* but couples the two through *o*, which exhibits oscillatory behavior for physiological values of *c*. To directly relate *c* to *c*s, we average *o* over one full cycle of its oscillation, the values of which are fully determined by *c* through the PA subsystem, as illustrated in the previous section. The nullcline relation Equation 8 can thus be approximated and rewritten as

$$0=\langle c_{\infty }(c)\rangle -c_{\text{s}},$$ (9)

where

$$\langle c_{\infty }(c)\rangle \equiv \overset{2\pi }{\underset{0}{\int }}c_{\infty }(o^{*}(\theta ;c))\frac{\text{d}\theta }{2\pi }={\overset{-}{c}}_{\infty }+\overset{2\pi }{\underset{0}{\int }}e^{-bo^{*}(\theta ;c)}\frac{\text{d}\theta }{2\pi }.$$ (10)

**Figure 3.**
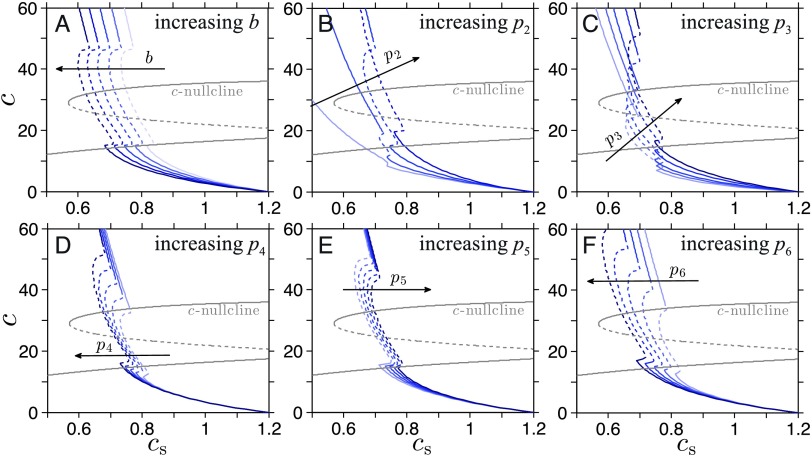
**Effect of changing parameters on *c*s-nullcline.** One of the six parameters that affect the *c*s-nullcline is varied over a range of values (from 80% to 120% of the reference values), and corresponding *c*s-nullclines are plotted. The dashed segment of the nullcline indicates the time-averaged value of *c*s over the limit cycle corresponding to the value of *c* (Equations 9 and 10). Darker colored curves (in the direction of the arrows) are associated with greater values of the corresponding parameter. When not varied, these parameters are set at the reference values: *t*_d_ = 1.44,*b* = 0.6,*p*_2_ = 15,*p*_3_ = 7.2,*p*_4_ = 0.05,*p*_5_ = 0.11, and *p*_6_ = 2.9. A) The value of *b* is varied from 0.48 to 0.71. B) The value of *p*_2_ is varied from 1.5 to 27. C) The value of *p*_3_ is varied from 3.6 to 7.92. D) The value of *p*_4_ is varied from 0.04 to 0.06. E) The value of *p*_5_ is varied from 0.09 to 0.14. F) The value of *p*_6_ is varied from 2.3 to 3.8.

The term o^*^(*θ*;*c*) represents the trajectory of *o*(*t*) with phase *θ* along the limit cycle of the PA subsystem defined by a given *c*. Therefore the *c*s-nullcline depends on the dynamics of the PA subsystem (Equations 3–4) and its parameters *b*,*p*_2_,*p*_3_,*p*_4_,*p*_5_, and *p*_6_. We can now vary one of these parameters while fixing the others to (Kim et al., 2016; Walker et al., 2010) *b* = 0.6,*p*_2_ = 15,*p*_3_ = 7.2,*p*_4_ = 0.05,*p*_5_ = 0.11, and *p*_6_ = 2.9, as illustrated in Figure 3. The part of the *c*s-nullcline that is approximated by averaging $c_{\infty }(o)$ over a full period of the limit cycle is indicated by dashed segments.

Figure 3A shows that increasing *b* shifts the *c*s-nullcline to the left in the (*c*s,*c*)-plane. This shift toward lower values of stored CRH, *c*s, is expected, because higher *b* corresponds to stronger cortisol-induced suppression of the CRH synthesis. On the other hand, as shown in Figures 3B and3C, increasing *p*_2_ or *p*_3_ lengthens the oscillatory regime of the *c*s-nullcline and shifts it toward the right. Increasing *p*_4_ elongates the oscillatory regime, while increasing *p*_5_ shrinks it. In both cases, a relatively small horizontal shift of the *c*s-nullcline is observed (Figures 3D and3E). Finally, increasing *p*_6_ increases the upper limit of *c* of the oscillatory branch and shifts it to smaller values of *c*s, as shown in Figure 3F.

How the long-term behavior of the HPA axis varies as physiological parameters change is now mapped out. We can use these results to predict what long-term changes would emerge under various pathological conditions, as illustrated in the next section. Note that many physiological disruptions may alter more than one parameter and that changing multiple parameters simultaneously can lead to a qualitatively different deformation of the *c*s- and *c*-nullcline. The effect of multiple parameter changes is beyond the scope of this article. However, we can easily extend our analysis to address the issue by altering all the parameters of interest when generating the nullclines.

## Results and Discussion

In the previous section, we have shown that the long-term dynamics of the system are determined by the crossing of the *c*- and *c*s-nullclines defined by Equations 6 and 9. Thus we only need to understand how the intersections change upon varying model parameters to study how alterations in parameters will affect the system. As a demonstration, we will predict how the parameter change observed in cerebral ischemia, a condition in which there is insufficient blood flow to the brain, would affect cortisol levels using the nullcline analysis from the previous section. Our predictions will be then compared to experimental observations, showing good agreement between them.

Finally, having studied how the HPA axis responds to parameter changes, we use our model to better understand a series of pharmacological challenge tests used to assess HPA function in PTSD. We reexamine some current interpretations by comparing them to our predictions and uncover unforeseen intricacies underlying the response of the HPA axis. Our analysis allows us to present novel, alternative interpretations of the observed challenge test experimental data. Indeed, this is one of the main virtues of mathematical models: They allow for probing complex, nonlinear dependencies and for crafting nontrivial predictions. Although the model presented in Equations 1–5 can be used to study any condition or experimental protocol that involves parameter alterations in the HPA axis, we focus, in this article, on experiments related to PTSD.

### Physiological Changes

In a previous study on male rats (Patricia, Raymond, Milot, Merali, & Plamondon, 2014), a long-lasting (30 days) increase in the expression of CRH receptor of Type 1 (CRHR-1) was observed in the PVN after global cerebral ischemia. The receptor is a G-protein coupled receptor that stimulates CRH secretion of the PVN neurons by increasing intracellular Ca^ +2^ (Hazell et al., 2012). Consequently, the maximum of the CRH secretion rate is increased, which corresponds to higher values of *q*_0_ in our model. Figure 2A implies that an increase in *q*_0_ will shift the *c*-nullcline leftward in the (*c*s,*c*)-plane, which in turn will shift the intersection of the *c*s- and *c*-nullclines toward higher *c* values. This upward shift predicts elevated CRH and cortisol level after ischemia, consistent with reports in Patricia et al. (2014). This example demonstrates how the nullcline structure analysis can provide insights in analyzing experimental observations. We will look at experimental results on PTSD subjects in the following subsections in a similar manner.

In particular, we use results from the previous section to revisit the hypothesis (Yehuda et al., 2004a; Yehuda, Golier, Yang, & Tischler, 2004b) of cortisol exerting an enhanced negative feedback on the pituitary under PTSD, which is commonly invoked to explain the low cortisol levels observed in PTSD patients. We can mathematically model the enhanced negative feedback of cortisol by increasing *p*_2_ in Equation 3, which controls the sensitivity of the feedback function *f*_*a*_(*or*) = 1/(1 + *p*_2_(*or*)). Our results are shown in Figure 3B and indicate that increasing *p*_2_ shifts the *c*s-nullcline toward the right, away from the lower branch of the *c*-nullcline. We thus predict that enhanced inhibition of the pituitary by cortisol will typically *increase* cortisol levels. Parameter changes reflecting enhanced inhibition in this class of models cannot explain the lower cortisol levels often associated with PTSD. However, earlier models (S. Gupta et al., 2007; Kim et al., 2016) and our current work allow for bistable steady states to arise, providing an alternative mechanism by which PTSD subjects may manifest low baseline cortisol. In particular, low cortisol diseased states arise as *a transition* from one stable state to another rather than from permanent physiological changes. To further investigate if this perspective is consistent with other experimental observations, we revisit a study in which dexamethasone (DEX) suppression was tested on PTSD patients.

### Dexamethasone Suppression Test (DST)

The dexamethasone suppression test (DST) is a pharmacological challenge test typically used to identify the cause of abnormal cortisol levels observed in diseases such as Cushing syndrome. In DST, a cortisol analogue (dexamethasone, DEX) is used to probe the negative feedback of cortisol on ACTH secretion in the pituitary. In particular, some studies have used DST to test whether lower basal cortisol levels in PTSD result from an enhanced negative feedback of cortisol on pituitary activity. DEX is a synthetic glucocorticoid compound that suppresses ACTH secretion, and subsequently cortisol secretion, when it binds to glucocorticoid receptors (GR) in the pituitary (Cole, Kim, Kalman, & Spencer, 2000). In DEX suppression studies, cortisol levels are measured pre-DEX and post-DEX and used to calculate the percentage suppression of cortisol, defined as

$$s=\frac{\text{pre-DEX cortisol}-\text{post-DEX cortisol}}{\text{pre-DEX cortisol}}\times 100.$$ (11)

The mean percentage suppression of cortisol *s* was shown to be greater in PTSD subjects (*s* = 83*%*) than in the control group without PTSD (*s* = 74*%*Yehuda et al., 2004a). The difference in *s* was interpreted as due to heightened sensitivity of the negative feedback of cortisol in the pituitary (Stein, Yehuda, Koverola, & Hanna, 1997; Yehuda et al., 2004b). This interpretation implicitly assumes that the suppression effect of cortisol (and dexamethasone) is directly proportional to its concentration. The linear relationship is convenient but neglects the contribution of other components of the system that can interact with the suppression activity to yield a more complex, possibly nonlinear relationship. We use our mathematical model to examine how exogenous DST affects the dynamics of the HPA axis. We model DEX administration by replacing *o*(*t*) with *o*(*t*) + *o*exo(*t*) in Equations 3 and 4 to yield

$$\frac{\text{d}c_{\text{s}}}{\text{d}t}=\frac{c_{\infty }(o)-c_{\text{s}}}{t_{c}},$$ (12)

$$\frac{\text{d}c}{\text{d}t}=q_{0}I(t)h(c_{\text{s}})+g_{c}(c)-q_{2}c,$$ (13)

$$\frac{\text{d}a}{\text{d}t}=\frac{c}{1+p_{2}((o+o_{\text{exo}}(t))r)}-p_{3}a,$$ (14)

$$\frac{\text{d}r}{\text{d}t}=\frac{{\left((o+o_{\text{exo}}(t))r\right)}^{2}}{p_{4}+{\left((o+o_{\text{exo}}(t))r\right)}^{2}}+p_{5}-p_{6}r,$$ (15)

$$\frac{\text{d}o}{\text{d}t}=a(t-t\text{d})-o.$$ (16)

Here *o*exo(*t*) denotes the concentration of circulating DEX converted to equivalents in cortisol concentrations based on the relative potency of DEX from Steven (1997). Note that we do not include DEX in Equation 12 because it was shown that DEX retention is much lower in the brain (Steckle, Kalin, Reul, & Hans, 2005) and that DEX does not affect GR in brain tissue (Cole et al., 2000). In a typical DEX challenge test, cortisol levels are measured in the morning (8:00 a.m.) to obtain basal pre-DEX cortisol levels. DEX is then orally administered, typically at night (11:00 p.m.), and post-DEX cortisol levels are measured again the following morning (8:00 a.m.). We assume circulating DEX levels follow a simple pharmacokinetic law:

$$\frac{\text{d}o_{\text{exo}}}{\text{d}t}=\Pi_{\text{o}}(t)-p_{7}o_{\text{exo}},$$ (17)

where *p*_7_ represents the decay rate of DEX relative to the decay rate of cortisol. For simplicity, we use a rectangle function for *Π*_o_(*t*). The width (30 min) of *Π*_o_(*t*) is estimated based on the at-peak concentration of DEX (Perez, Rogers, Smith, & Weisman, 1998) and the height (2 in nondimensionalized units of *o*) is set to match the dosage used in the experiment (Yehuda et al., 2004a). Based on the half-life of DEX (∼ 240 min; Perez et al., 1998) and cortisol (∼ 7.2 min; Lightman et al., 2008), we set *p*_7_ = 0.03.

**Figure 4.**
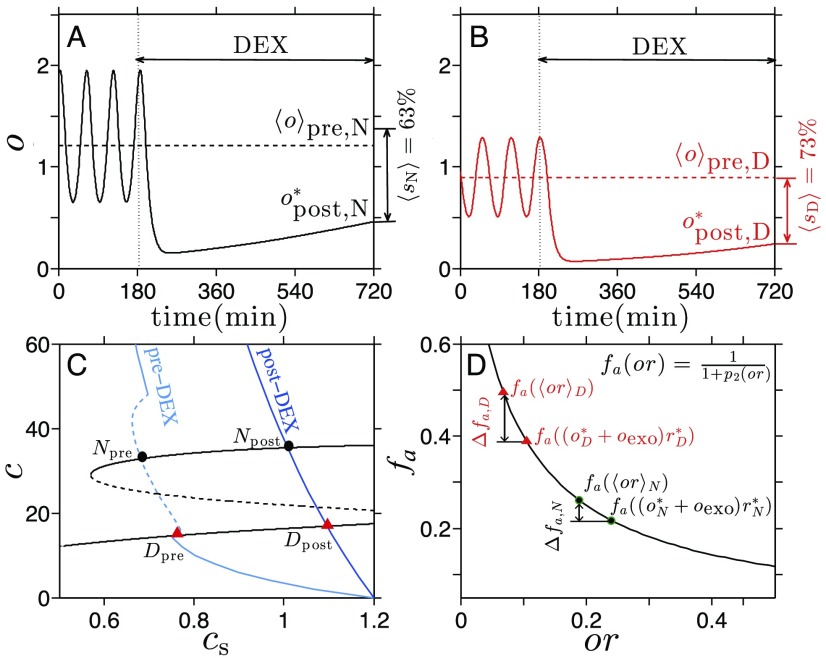
**Numerical simulation of DEX challenge test on normal and PTSD subjects.** A) Numer ical simulation of cortisol response in a “normal” subject during DEX suppression test. The aver age percentage suppression of cortisol in this scenario is 〈*s*_*N*_〉 = 63*%*. B) Numerical simulation of cortisol response in a PTSD subject during DEX suppression test. Here the average percentage suppression of cortisol is 〈*s*_*D*_〉 = 73*%*, significantly greater than that of a normal subject. C) Inter sections of the *c*-nullcline with pre-DEX (light blue) and post-DEX (blue) *c*s-nullclines. Normal and PTSD subjects are represented as black circles and red triangles, respectively. D) The values of *f*_*a*_(*or*) pre- and post-DEX treatment are plotted for normal (black circles) and PTSD states (red triangles).

The numerical solutions of cortisol levels during DEX suppression test as modeled by Equations 12–16 are shown in Figures 4A and4B for normal and PTSD subjects, respectively. The parameters used in both figures are identical: Normal and PTSD subjects are characterized solely by which one of the two stable states they initially reside in (*c*s,*c*) space. Initial (*c*s,*c*) values are plotted in Figure 4C and labeled *N*_pre_ and *D*_pre_ for normal and PTSD subjects, respectively. We take the average of *o* over a full cycle of oscillation to estimate pre-DEX cortisol values 〈*o*〉_pre,*N*_ = 63*%* and 〈*o*〉_pre,*D*_ = 73*%* for normal and PTSD subjects. These predicted values are in qualitative agreement with the experimentally reported percentage suppression *s*_N_ = 74*%* and *s*_D_ = 83*%* for normal and PTSD subjects (Yehuda et al., 2004a). Owing to the oscillatory behavior of cortisol in the basal sate, the percentage suppression depends on the phase of the oscillation at the time of pre-DEX measurement. For example, if the pre-DEX measurement time is set near the peak of the oscillations, our model predicts *s*_N_ = 76*%* for normal subjects and *s*_D_ = 81*%* for PTSD subjects. For a more accurate comparison between our predictions and data, the *timing* of DEX administration with respect to the phase of the oscillating cortisol levels should be carefully controlled in experiments.

To understand the behavior of our HPA axis model under DEX administration, we observe how *o*exo(*t*) affects the (*c*s,*c*)-nullcline structure. Because *o*exo(*t*) contributes only to the PA subsystem (Equations 3–5), it only affects the *c*s-nullcline. The latter is shown in Figure 4C (dark blue) near the time of measurement, 9 hours from DEX administration. Normal and diseased states initially resting at the intersection of the *c*s- and *c*-nullclines slowly evolve toward the shifted intersections (labeled *N*_post_ and *D*_post_ for normal and PTSD subjects, respectively) defined by the new *c*s-nullcline under DEX administration.

We use the decrease in *f*_*a*_(*or*) = 1/(1 + *p*_2_(*or*)) in Equation 3 due to DEX administra tion as a measure of suppression of pituitary activity under the challenge test. Recall that *f*_*a*_(*or*) is a modulating factor of the ACTH production rate and represents the negative feedback of the cortisol on the pituitary. For the pre-DEX value of *f*_*a*_(*or*), we take the period-averaged value of *or*, denoted 〈*or*〉_*N*_ and 〈*or*〉_*D*_ for normal and diseased initial states, respectively. Note that the new shifted *c*s-nullcline under DEX administration is not associated with oscillatory behavior and that the new intersections represent nonoscillating equilibria. The cortisol levels depicted in Figures 4A and4B confirm that cortisol oscillations cease under DEX suppression, which has not yet been experimentally tested. The nonoscillating equilibrium values for *o* and *r* are denoted $o_{\alpha }^{*}$ and $r_{\alpha }^{*}$, where *α* = *N*,*D* indicate normal and PTSD states. In Figure 4C, pre-DEX values of *f*_*a*_(〈*or*〉_*α*_) and post-DEX values of $f_{a}((o_{\alpha }^{*}+o_{\text{exo}})r_{\alpha }^{*})$ are plotted for normal (*α* = *N*) and PTSD (*α* = *D*) subjects. The decreases in *f*_*a*_(*or*) denoted as *Δf*_*a*,*α*_ in Figure 4D indicate that suppression is greater for PTSD subjects than for normal subjects, despite the comparable change in *or* values. This is because of the form of *f*_*a*_(*or*): Decreases in *f*_*a*_(*or*) due to increases in *o* are greater for lower initial values of *or*. In other words, the state with low initial *or* value will experience greater suppression as *o* increases to *o* + *o*_exo_ because *f*_*a*_(*or*) is convex (*f*_*a*_(*or*)″ > 0) and decreasing (*f*_*a*_(*or*)*′* < 0) for *o* ≥ 0 and *r* ≥ 0.

Our model provides an additional interpretation of DEX administration results in PTSD patients. The enhanced suppression effect on cortisol may be due to the *intrinsic* dynamics rather than parametric changes; within our model, the negative feedback effect is dependent on the state of the system at the time of DEX administration, which in turn depends on dynamics and history of the system. Thus increased suppression of cortisol levels in PTSD subjects during DEX administration may simply indicate that PTSD subjects were in the low cortisol state *before* the test rather than implying that permanent changes had occurred in their system in the course of developing PTSD.

This alternate explanation has direct implications for how one should design therapeutic protocols for PTSD or other stress-related disorders associated with cortisol disruption. Under the enhanced negative feedback hypothesis, therapies or medications would attempt to lower the pituitary sensitivity to cortisol to bring it back to normal levels. Our model shows that this “straightforward” approach would fail and suggests that therapies should instead focus on devising appropriate perturbations to the system. For example, applying externally controlled inputs *I*(*t*) could induce transitions back to the normal stable state, as shown in Kim et al. (2016). This framework is consistent with current recommendations for treatment of stress disorders via exposure therapy and cognitive-behavioral techniques in which an individual is reexposed to trauma or stress in a controlled way.

### ACTH Stimulation Test

In this section, we consider the ACTH stimulation test typically used to diagnose conditions associated with insufficient adrenal activity. Cortisol levels are measured after the administration of cosyntropin, a synthetic derivative of ACTH. Exogenous ACTH stimulates cortisol secretion to the same extent of endogenous ACTH and thus effectively increases *a*(*t*) in our model. As in the analysis of the DEX suppression test, we can model cosyntropin administration by replacing *a*(*t*) with *a*(*t*) + *a*exo(*t*), where *a*exo(*t*) denotes the concentration of the cosyntropin in circulation.

In a previous study (Radant et al., 2009), an ACTH stimulation test was administered to PTSD and normal subjects to measure potential differences in adrenal gland response between the two groups. It was hypothesized that a bolus of cosyntropin would lead to a *smaller* pulse of cortisol secretion in PTSD patients due to hyporeactivity of their adrenal glands. Adrenal hyporeactivity would also suggest lower baseline cortisol levels, consistent with a number of observations (Yehuda et al., 2004a,s). Surprisingly, the main experimental finding was that cortisol response to cosyntropin *was not* significantly altered in PTSD subjects. Moreover, the baseline cortisol levels observed under PTSD were actually slightly higher than normal (Radant et al., 2009). It was thus concluded (Radant et al., 2009) that either adrenal reactivity is not altered in PTSD patients or that potential alterations do not affect HPA dynamics.

This interpretation relies on the intuition that a proportional relationship exists between adrenal reactivity and cortisol response so that upon stimulation, in this case by cosyntropin, any adrenal hyporeactivity in PTSD subjects would lead to smaller cortisol increases. This picture would be valid if the stimulating activity of ACTH in the adrenal gland were isolated from other ACTH interactions within the HPA axis. However, cortisol suppresses endogenous ACTH secretion in the pituitary, which in turn indirectly reduces cortisol secretion. In addition, glucocorticoid receptor (GR) concentration is coupled to cortisol through the dependence on *or* in Equation 4 and thus influences the negative feedback in the pituitary. As a result of these various nonlinear interactions, particularly those embodied by *g*_*r*_(*or*) and *f*_*a*_(*or*), cortisol response to ACTH stimulation is more complex than a simple proportionality relationship. We now use our model to explore these nonlinear interactions and develop a more nuanced interpretation of the experimental observations.

**Figure 5.**
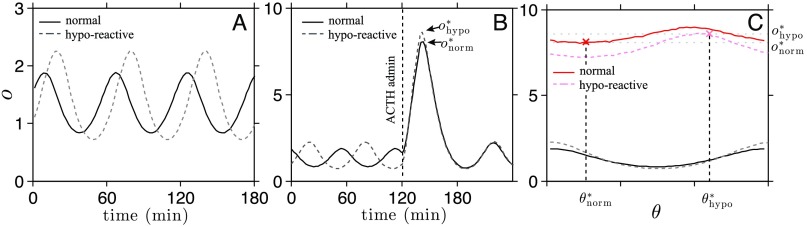
**Numerical solutions of ACTH stimulation test.** A) The oscillating stable state of the system with normal (solid) and hyporeactive (dashed) adrenal gland sensitivity is plotted. The non dimensionalized *o* is scaled by the same factor (the normal adrenal sensitivity) in both cases for direct comparison. Details of the scaling of the state variables are provided in Kim et al. (2017, Appendix A). The hyporeactive subject with *p*_2_ and *p*_4_ adjusted to represent lower adrenal sensitivity exhibited slightly higher basal cortisol levels. B) Cortisol response to exogenous ACTH administration is plotted for normal (solid) and for hyporeactive (dashed) adrenal sensitivity. The phase of the oscillation at the time of administration was different in each simulation. C) The peak cortisol levels reached during exogenous ACTH administration are plotted as a function of the phase of the intrinsic oscillations at the time of ACTH administration for normal (solid red) and hyporeactive (dashed red) subjects. The phase and the peaks shown in B are marked in the plot as an example. The maximum peak cortisol level of the hyposensitive subject and the minimum peak cortisol level of the hyporeactive subject are both approximately *o* = 8.

In particular, we contrast and compare our results to the previously described experimental data and show that, indeed, upon taking into consideration the full dynamics of the HPA axis, laboratory observations (Radant et al., 2009) *may be consistent* with the hypothesis of reduced adrenal reactivity in PTSD subjects, in contrast to the original interpretation. Within our model, adrenal gland reactivity to ACTH determines the parameters *p*_2_ and *p*_4_ in Equations 1–5 (refer to Kim et al., 2017, Appendix A, for details). To model hyporeactivity, we adjust both parameters to reflect a 10% decrease in the adrenal gland reactivity in PTSD subjects. The resulting numerical solutions are plotted in Figure 5A and show that basal cortisol levels are indeed *increased* in the basal state of PTSD subjects, consistent with the baseline cortisol measurements in Radant et al. (2009). This result emphasizes that the simple intuition of a direct relationship between adrenal reactivity and cortisol response can be misleading.

To now describe the response of cortisol under an ACTH stimulation test, we modify our model by rewriting Equation 5 as

$$\frac{\text{d}o}{\text{d}t}=a(t-t\text{d})+a_{\text{exo}}(t-t_{\text{d}})-o,$$ (18)

where *a*exo(*t*) denotes the concentration of exogenous cosyntropin in circulation. We model the dynamics of *a*exo(*t*) using a pharmacokinetic description similar to that used for DEX:

$$\frac{\text{d}a_{\text{exo}}}{\text{d}t}=\Pi_{a}(t)-p_{8}a_{\text{exo}}.$$ (19)

Here *p*_8_ represents the clearance rate of cosyntropin scaled by the clearance rate of cortisol and *Π*_*a*_(*t*) is a rectangle function. We set *p*_8_ = 1.7 based on the 4.1-min half-life of exogenous ACTH (Matsuyama, Ruhmann-Wennhold, Johnson, & Nelson, 1972). We compare cortisol’s response to cosyntropin administered near the nadir of its ultradian oscillation for hyporeactive and near the peak for normal adrenal glands in Figure 5B. In this case, the response of a hyporeactive subject is higher than that of a normal subject. To study the dependence of cortisol response on the timing of cosyntropin administration, in Figure 5C, we plot the peak levels of cortisol under cosyntropin administration against the phase of the oscillation at which the administration took place. Overall, cortisol response is predicted to be slightly *greater* in normal subjects, with the maximum peak response of a hypo-sensitive subject (dashed red in Figure 5C) comparable to the minimum peak response of a normal subject (solid red in Figure 5C). This prediction implies that, depending on the phase of the intrinsic oscillation at the time of cosyntropin administration, the response of a hyporeactive adrenal gland could be greater than normal. However, the corresponding *mean* cortisol response should be lower if a sufficiently large sample size is used without controlling for the phase of cortisol at the time of cosyntropin administration.

Our model predicts that reduced adrenal reactivity to ACTH will *increase* baseline cortisol levels. This implies that the experimental result reported in Radant et al. (2009) may indeed reflect a reduced adrenal reactivity in PTSD subjects, amending previous interpretations. The increase in cortisol response seen among PTSD patients can also vary depending on the timing of cosyntropin administration. For example, the relative increase in cortisol is greatest upon administering cosyntropin at the nadir of the cortisol oscillation, as seen in Figures 5B and5C. Note that if experiments on normal and PTSD individuals are not phase matched, the maximum response of a normal subject may be greater than the minimal response of a PTSD subject. Thus phase matching would be required to remove confounding effects arising from the timing of ACTH/cosyntropin administration. For example, the relative increase in cortisol is greatest upon administering cosyntropin during the increasing phase near the nadir of cortisol’s intrinsic oscillation, as seen in Figure 5C. Note that if experiments on normal and PTSD individuals are not phase matched, the response of a normal subject may be less than the response of a PTSD subject, as observed from the experiment. Because the sample size used in the experiment (Radant et al., 2009) was small (*n* = 8 subjects for PTSD and *n* = 9 subjects for control), the increased cortisol response among PTSD subjects may be explained as a confounding effect arising from the timing of ACTH/cosyntropin administration. Phase matching would be required for a proper interpretation of the measurement.

### Predictions for a New Two-Stage Challenge Test

In the previous section, we reexamined the hypothesis that changes in physiological parameters (specifically *p*_2_) can result in a greater suppression of pituitary activity by dexamethasone or cortisol. This enhanced negative feedback has been proposed as the cause for lower basal cortisol levels in PTSD subjects. Using our model and analyses, we presented an alter native hypothesis based on the nonlinear interactions in our dynamical system. Here we outline new experiments that could be used to further evaluate and distinguish these two hypotheses. If the negative feedback of cortisol on the pituitary is enhanced in PTSD subjects, their response to stressors should *be reduced*, especially when the pharmacological suppression is in effect. One way to further probe the system is to combine a nonpharmacological, psychological stressor with the DST. The ensuing cortisol response can be used to probe the purported enhanced negative feedback on the pituitary. The new challenge test consists of two stages: (a) Dexamethasone is administered to suppress the pituitary activity and (b) a psychological stressor applied during the suppression is in effect.

Physiological stressors are processed in various regions of the brain, and the information is transferred to the PVN neurons via synaptic inputs. Changes in the synaptic input can be represented by a perturbation in the *I* term in Equation 2 in the form *I*(*t*) = *I*_0_ + *I*_ext_(*t*), where *I*_ext_(*t*) represents the change in synaptic input received by the PVN neurons.

**Figure 6.**
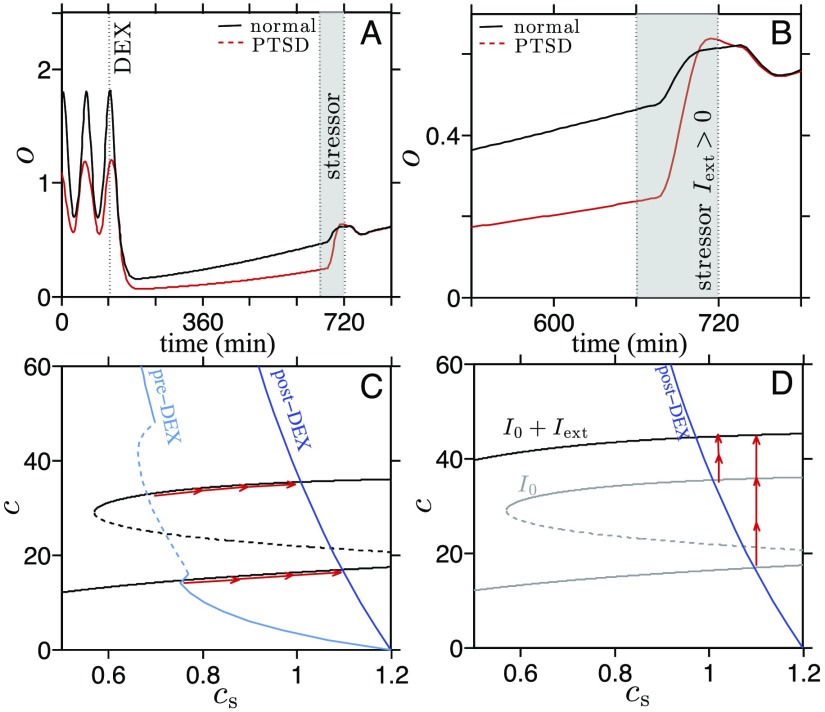
**Numerical solutions of a combined two-step challenge test.** We propose a new challenge test in which a nonpharmacological stress challenge is given after DEX administration. A) The cortisol responses of normal and diseased state systems to a nonpharmacological stressor *I*_ext_(*t*) at the typical time (9 hours) of post-DEX measurement in DST (shaded region). The response is greater in the system with lower cortisol level (dashed red, PTSD group) than the control (solid black) despite the larger suppression induced by DEX prior to the stressor. B) A close-up of A shows that the peak cortisol level in PTSD subjects surpasses that in normal subjects during the external stress. C) Nullcline structure during DEX suppression is similar to the one in Figure 4C before the external stressor *I*_ext_ is applied. The *c*s-nullcline jumps immediately after DEX administration (light blue to dark blue) and relaxes very slowly back to its original position. The stable points slide along the upper and the lower branches of the *c*-nullcline toward the new intersection with the temporarily shifted *c*s-nullcline (dark blue). D) The *c*-nullcline is shifted leftward and upward during application of the stressor *I*_ext_. The states on the upper and lower branch of the original *c*-nullcline quickly move toward the new *c*-nullcline (red arrows). The increase during the shift in *c* (and subsequently in *o*) is greater for the PTSD state.

During the second stage of our proposed test, we assume *I*_ext_(*t*) to be a positive constant when the external (psychological) stressor is on, and zero while off. In our thought experiment, we turn *I*_ext_ on for 60 min starting 9 hours after DEX administration, when post-DEX cortisol levels are usually measured. As can be seen in Figures 6A and6B, our model predicts cortisol response to be generally greater in the low-cortisol PTSD state than in the normal state, in contrast to the enhanced negative feedback hypothesis. Moreover, the peak of cortisol under PTSD can surpass that of cortisol under normal conditions if *I*_ext_ is sufficiently large.

To understand this unexpected “reversed” phenomenon, consider the nullcline structure during DEX administration and after the stressor is applied (Figures 6C and6D). Upon turning the stressor on, the *I*_ext_ perturbation increases the secretion rate of CRH of the PVN neurons, effectively changing *q*_0_ in Equation 2. We have previously shown that increasing *q*_0_ shifts the upper branch of the *c*-nullcline and moves the bistable regime toward the left in the (*c*s,*c*)-plane, as shown in Figure 2A. For a stressor with *I*_ext_ = 0.5, the *c*-nullcline is shifted so that the PTSD state on the lower branch is no longer in the bistable regime in the (*c*s,*c*)-plane and, consequently, the PTSD state *jumps* to the upper branch and relaxes toward the only available steady state, approximated by the intersection of the two nullclines in Figure 6D. Meanwhile, the normal state residing on the upper branch of the *c*-nullcline also jumps to the shifted nullcline, but the size of the jump is significantly *smaller* compared to the jump from the lower branch (as seen in Figure 6D).

The proposed two-step challenge protocol shows discrepancies between the results of our model and the enhanced negative feedback hypothesis. The increase in cortisol response in a subject with lower basal cortisol level *cannot* be explained by an altered negative feedback strength, while it can be understood as a natural consequence of the changing dynamical structure of the system owing to perturbations in the parameter. The experiment also eliminates the confounding effect of measurements taken from hourly oscillating cortisol levels.

## Summary and conclusions

The HPA axis is a dynamical system continuously evolving to meet changing physiological needs and environmental stimuli. Even at equilibrium, key hormones, such as cortisol and ACTH, exhibit ultradian oscillations. To accurately interpret the response of the HPA axis to internal, anatomical changes or external inputs, such as the injection of exogenous hormones, we need to understand how the response depends on the state of the system itself and the interplay between its different components. To this end, we developed a mathematical model of HPA dynamics where changes in parameter values and exogenous sources of key hormones could be explicitly included. Of particular importance is the understanding of how pharmacological intervention might affect the long-term dynamics of the HPA axis. Our model is useful in this respect because the effect of medication, trauma, or disease can be mapped onto changes in given parameters. We thus performed a parameter sweep and were able to predict possible modifications to long-term behaviors induced by corresponding pharmacological challenge tests.

Measurements taken during an ACTH stimulation test have shown higher baseline cortisol levels in PTSD subjects and unchanged cortisol responses to exogenous ACTH administration (Radant et al., 2009). This result was interpreted as evidence against altered adrenal reactivity in PTSD subjects. Upon incorporating the altered adrenal gland reactivity into our model as a parameter change, we found that data from Radant et al. (2009) *can* be explained by adrenal hyporeactivity in PTSD.

Our simulations show that cortisol levels in subjects with hyporeactive adrenal glands can increase by amounts that are comparable to the increases observed in normal subjects upon the administration of exogenous ACTH. We believe that the phase of the intrinsic oscillations in cortisol at the time of exogenous ACTH administration should be controlled for a more accurate interpretation of experiments.

The most well known feature of HPA dysfunction in PTSD patients is the low secretion of cortisol. The current viewpoint is that low cortisol levels are a consequence of an enhanced negative feedback in the HPA axis (Bremner et al., 2007; Yehuda et al., 1996). This conclusion is based on DEX suppression tests that show a greater percentage suppression of cortisol among PTSD subjects. Extending our model to include DEX administration, we have provided an additional mechanism to explain low cortisol levels in PTSD, namely, that it arises as a feature of an alternate stable state of the dynamical system. The diseased low-cortisol stable state exhibits relatively greater suppression of cortisol *without* enhancing the sensitivity of the negative feedback of cortisol in the pituitary and without the need to invoke externally induced parameter changes. Thus enhanced cortisol suppression is an inherent feature of a stable state in our bistable model.

Our model can help us understand the many ways trauma might dysregulate cortisol dynamics and identify subgroups of PTSD patients that may require different treatment approaches. For instance, one could measure specific parameters for subjects within one of the subgroups that showed a significant difference in cortisol level (Meewisse et al., 2007) and verify whether the associated nullcline structure would allow for bistability. If so, our model supports possible treatments in the form of appropriate external inputs to the system to induce the transition between the stable states. To the contrary, if bistability does not arise, treatment protocols should focus on adjusting the parameter values to correct the dynamics.

Although our model has provided a mechanistic description of the HPA axis behavior under two distinct pharmacological challenge tests, it does not provide a direct explanation for the variability observed in baseline cortisol levels in PTSD patients. One possibility is that the discrepancy merely reflects different stages or effects of disruptions in the HPA axis induced by exposure to trauma.

The literature on PTSD has been driven by diagnostic criteria that rely heavily on nonquantitative and subjective self-reports. This is also true with research on neuroendocrine alterations in PTSD, where the definition of PTSD often failed to take into account important factors, such as gender and type of trauma. Such issues have likely contributed to conflicting reports on how basal cortisol levels are affected by PTSD. Our mathematical model provides a framework to help interpret how external perturbations, such as pharmacological challenge tests, lead to abnormal dynamics and long-term behavior. Analysis of our model allows us to characterize, more mechanistically, PTSD by identifying specific components of the HPA axis that can be affected by trauma or medication.

## AUTHOR CONTRIBUTIONS

Lae Un Kim, Maria R. D’Orsogna, and Tom Chou designed the research. Lae Un Kim per formed the analysis and simulations. Lae Un Kim, Maria R. D’Orsogna, and Tom Chou wrote and edited the manuscript.

## FUNDING INFORMATION

This work was supported by the Army Research Office via grant W911NF-14-1-0472 and the NSF through grant DMS-1516675.

## ACKNOWLEDGMENT

The authors also thank the late Professor T. Minor for insightful discussions.
